# Neural network enabled wide field-of-view imaging with hyperbolic metalenses

**DOI:** 10.1515/nanoph-2025-0354

**Published:** 2025-09-18

**Authors:** Joel Yeo, Deepak K. Sharma, Saurabh Srivastava, Aihong Huang, Emmanuel Lassalle, Egor Khaidarov, Keng Heng Lai, Yuan Hsing Fu, N. Duane Loh, Arseniy I. Kuznetsov, Ramon Paniagua-Dominguez

**Affiliations:** Institute of Materials Research and Engineering (IMRE), Agency for Science, Technology and Research (A*STAR), 2 Fusionopolis Way, Innovis #08-03, Singapore 138634, Republic of Singapore; NUS Graduate School for Integrative, Sciences and Engineering Programme, 37580 National University of Singapore, 119077, Singapore, Republic of Singapore; Department of Physics, National University of Singapore, 117551, Singapore, Republic of Singapore; Institute of Microelectronics (IME), Agency for Science, Technology and Research (A*STAR), 2 Fusionopolis Way, Innovis #08-02, Singapore 138634, Republic of Singapore; Department of Biological Sciences, National University of Singapore, 117557, Singapore, Republic of Singapore

**Keywords:** flat optics, metalenses, neural network, deconvolution, imaging

## Abstract

The ultrathin form factor of metalenses makes them highly appealing for novel sensing and imaging applications. Amongst the various phase profiles, the hyperbolic metalens stands out for being free from spherical aberrations and having one of the highest focusing efficiencies to date. For imaging, however, hyperbolic metalenses present significant off-axis aberrations, severely restricting the achievable field-of-view (FOV). Extending the FOV of hyperbolic metalenses is thus feasible only if these aberrations can be corrected. Here, we demonstrate that a Restormer neural network can be used to correct these severe off-axis aberrations, enabling wide FOV imaging with a hyperbolic metalens camera. Importantly, we demonstrate the feasibility of training the Restormer network purely on simulated datasets of spatially-varying blurred images generated by the eigen-point-spread function (eigenPSF) method, eliminating the need for time-intensive experimental data collection. This reference-free training ensures that Restormer learns solely to correct optical aberrations, resulting in reconstructions that are faithful to the original scene. Using this method, we show that a hyperbolic metalens camera can be used to obtain high-quality imaging over a wide FOV of 54° in experimentally captured scenes under diverse lighting conditions.

## 1 Introduction

Metasurfaces have emerged as a transformative technology in optics due to their potential to replace, or even outperform, traditional optical components with ultra-thin, multi-functional ones. Within the field, the metasurface counterparts of traditional lenses (so-called metalenses) are particularly attractive as these are the most ubiquitous elements in optical systems, usually taking the vast majority of space and weight. Unlike traditional bulky lenses, metalenses utilize nanoscale structures to manipulate the fundamental properties of light (typically the phase) locally and abruptly, making them invaluable for applications in imaging, sensing, and optical metrology [1], [2]. The capability of metalenses to replicate complex phase profiles while remaining ultrathin also offers significant advantages over their bulky counterparts, where freeform optics is usually expensive and difficult to manufacture [3], [4], [5].

Within the different metalens designs explored by the community, the one that imparts a hyperbolic phase profile in the incident beam is particularly attractive as it is free from spherical (and any other spatial) aberrations when illuminated on-axis [5]. In addition, the focusing efficiency of these metalenses remains the highest demonstrated to date, making them commonly used for light-focusing applications, including those requiring high-numerical apertures (NA) [6], [7], [8], [9], [10], [11]. However, while the hyperbolic lens is theoretically diffraction-limited along the optical axis, it presents strong off-axis aberrations, translating into a point-spread function (PSF) that rapidly deteriorates as the angle of incident light departs from normal [12]. In an imaging experiment, this causes the resultant image to be aberrated with a spatially varying blur, which traditional deblurring methods such as the Wiener filter [13] cannot remove. As a consequence, these off-axis aberrations severely limit the usable field-of-view (FOV) of hyperbolic metalenses and, therefore, their use in imaging applications.

To circumvent this issue and expand the FOV of metalenses, the community has explored alternative phase profiles, such as the quadratic one [14], [15], [16], [17], [18], [19], [20], or multi-element configurations (doublets, triplets, or other lens arrays) [21], [22], [23], [24], [25]. While these are indeed able to provide a wide FOV (up to even 180° in some cases), they come at the cost of spherical aberrations and poor efficiencies (in the case of quadratic phase profiles) or fabrication complexity and overall system size (in the case of doublets or systems with an aperture).

As a result, part of the community is now turning their attention to the possibility of correcting this issue on the software side rather than the hardware one. In this regard, iterative deconvolution algorithms have been recently introduced to correct for such spatially varying aberrations [26]. These, however, are typically slow and prone to reconstruction artifacts [17]. These algorithms are also sensitive to noise and require precise calibration of the spatially-varying PSFs which is challenging in practical applications. Over recent years, deep-learning algorithms have been increasingly applied to remove aberrations from metalens images [27], [28], [29], [30], [31], [32], [33], [34], [35], [36]. Their fast inference speed, combined with robustness against noise and experimental errors, make them highly appealing and successful for metalens imaging postprocessing. However, many demonstrations of deep-learning deblurring are reference-based, requiring tedious curation of experimental datasets of measurement and ground truth pairs. This could also result in overfitting to specific imaging conditions, such as lighting, magnification, alignment, and other experimental parameters for which the experimental dataset was collected. As such, these trained networks would not be readily extended to deblur images under different imaging conditions.

Here, we present a neural network-enabled, reference-free hyperbolic metalens camera for wide FOV imaging. In particular, we employ a Restormer neural network to correct the severe off-axis aberrations of these type of lenses, ultimately enabling aberration-free imaging over 54°. By reference-free training, we mean that while the network is trained in a supervised manner, it does not require any experimentally acquired reference datasets, whether from external imaging systems or curated target images (e.g., pictures displayed on a screen). Instead, all training data are generated synthetically by simulating spatially varying blurred images using the eigenPSF method [26], which automatically provides the ground truth based on the physics of the imaging system itself. This eliminates the need for time-consuming curation of experimental datasets and also ensures that the trained network only removes optical aberrations without overfitting to specific imaging conditions. We demonstrate that our hyperbolic metalens camera delivers robust imaging performance in low-light conditions, during close-up photography, and under diverse lighting directions and occlusions.

## 2 Results

### 2.1 Design, fabrication and optical characterization of the metalens

**Figure 1:**
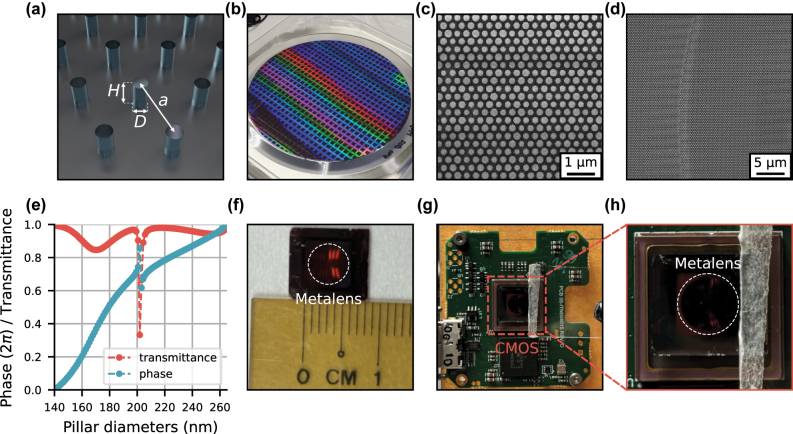
Fabrication of the hyperbolic metalens and the imaging setup. (a) Schematic of the hexagonal unit cell of the metalens with a lattice constant of *a* = 350 nm, consisting of cylindrical nanopillars with a height of *H* = 500 nm and diameters, *D*, ranging from 140 nm to 264 nm. (b) Optical image of a wafer with an array of metalenses patterned using deep UV immersion photolithography. (c, d) Scanning electron micrographs of the metalens depicting the patterned a-Si nanopillars on a glass substrate. (e) The simulated phase and transmittance of uniform a-Si nanopillar arrays with height of 500 nm and diameters ranging from 140 to 264 nm with a step size of 2 nm. (f) Optical image of the fabricated 5 mm diameter metalens. (g) Optical image of the hyperbolic metalens camera used in imaging, where the (h) metalens (white circle) is mounted directly in front of the CMOS detector (red square).

The hyperbolic metalens used in this work has a phase profile given by the expression

(1) $$\phi \left(r\right)=\frac{2\pi }{\lambda }\left(f-\sqrt{r^{2}+f^{2}}\right),$$

where *r* is the radial distance from the center of the lens, and *λ* and *f* are the design wavelength and focal length, respectively. The fabricated metalens has a diameter of *D* = 5 mm and *f* = 1.813 mm, designed at a working wavelength of *λ* = 850 nm with a numerical aperture of NA = 0.81. The (wrapped) hyperbolic phase profile was mapped using amorphous silicon (a-Si) nanopillars with a circular cross-section (to maintain polarization-insensitive response) on a fused silica substrate. These pillars are arranged in a hexagonal lattice (lattice constant of 350 nm) and have a fixed height of 500 nm and diameters in the range of 140–264 nm (Figure 1a–d). The simulated transmittance and phase as a function of the pillar diameter are also plotted in Figure 1e.

**Figure 2:**
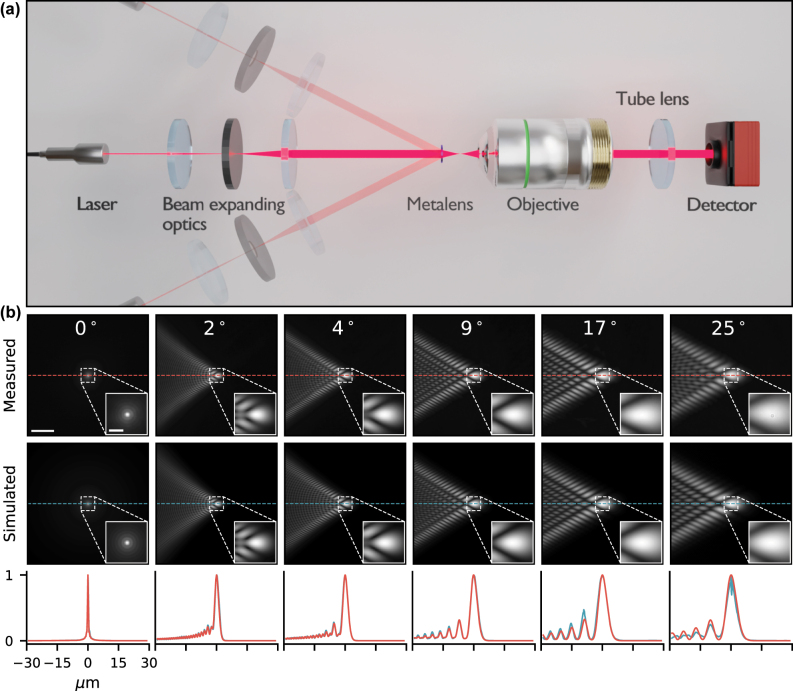
Characterization of the hyperbolic metalens. (a) Optical setup for angles of incidence dependent optical characterization of the hyperbolic metalens PSFs. The laser (850 nm wavelength) output isexpanded using two lenses and an aperture to distribute the intensity uniformly across the metalens. These are mounted on a rotating arm of a goniometer that enables different AOI illumination on the metalens. The metalens focuses the collimated laser at the focal plane, which is then imaged onto the CMOS detector using an objective lens (Olympus, MPLAPON100X) and a tube lens (150 mm focal distance). (b) (top) Experimentally measured PSFs at different angles of incidence compared to (middle) simulated PSFs for the hyperbolic metalens. We use a log-normalized colormap for better visualization of these PSFs. The scalebars (white) have sizes of 10 µm and 2 µm for the main image and its inset, respectively. (bottom) The horizontal line profiles of the measured (red) and simulated (blue) PSFs.

The samples are fabricated using a 12-inch, deep ultraviolet (UV) immersion photolithography scanner (see details in Methods) and optically characterized using a goniometric optical setup (Figure 2a). This characterization setup, which has a calculated magnification of 83.3 (resulting in an effective detector pixel size of 41.4 nm) allows imaging the PSFs of the fabricated hyperbolic metalens at various angles of incidence (AOI). Figure 2b compares these measurements against theoretical PSFs calculated with Fourier optics simulations (details in Supplementary information). As can be seen, there is a close match between the measured and simulated PSFs, with minor discrepancies likely attributed to fabrication errors.

### 2.2 Hyperbolic metalens camera

The hyperbolic metalens camera comprises only two components (Figure 1g and h): the metalens and a complementary metal oxide semiconductor (CMOS) detector (Thorlabs, Zelux-CS165MU), resulting in an ultra-compact design. The scene is illuminated with a light-emitting diode (LED) with a dominant wavelength of 850 nm (Thorlabs M850L3) and bandwidth of 30 nm (setup figure in Supplementary information). The hyperbolic metalens, mounted at a distance *f* = 1.813 mm from the detector, focuses the illuminated scene onto the sensor to form the image. In this work, we used only the detector’s central 512 × 512 pixels, corresponding to an angular field-of-view (FOV) of 54°, due to memory constraints during network training. Beyond this computational limitation, the practical FOV of our hyperbolic metalens camera is also restricted by physical factors: at larger angles, the PSFs eventually spread beyond the detector size, and exhibit weaker signal, making them difficult to capture and accurately model the physics of image formation for our eigenPSF method.

### 2.3 Restormer deblurring

**Figure 3:**
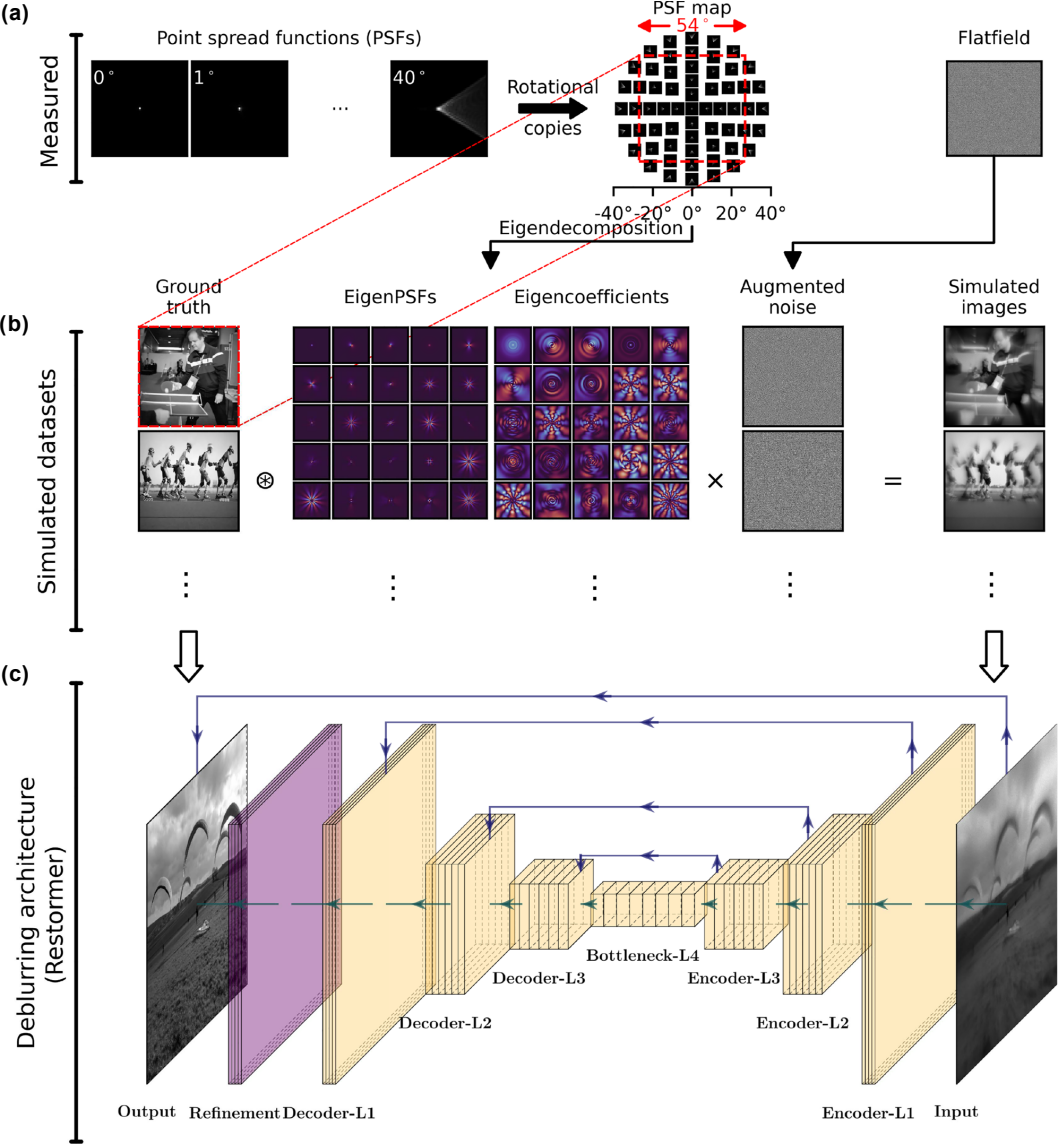
Schematic for computational deblurring using the Restormer architecture trained on eigenPSF-simulated images. (a) The hyperbolic metalens is characterized using the measured PSFs, and the flatfield records the noise profile of the imaging system. (b) Simulated images are obtained using the eigenPSF method to apply spatially varying blur on the ground truth dataset, and further corrupted by noise created by augmenting the flatfield measurement. (c) The Restormer architecture is used to deblur and denoise the images. Illustration created using PlotNeuralNet [39].

Figure 3 shows the schematic of our computational deblurring approach for hyperbolic metalens imaging. Using our imaging setup, we first measured the PSFs at different AOIs ranging from 0° to 40° (Figure 3a), with denser sampling at smaller angles to capture rapidly varying PSF shapes and sparser sampling at larger angles where the PSFs primarily grow in size (see Supplementary information). Note that these imaging PSFs are different from the measured PSFs depicted in Figure 2b as there is no external magnification in the imaging setup. These imaging PSFs are computationally rotated to populate a PSF map that covers the full extent of an image corresponding to an angular FOV of 54° as shown in Figure 3a. These spatially-varying PSFs are then eigendecomposed into spatially-invariant eigenPSF bases weighted by the corresponding eigencoefficients [26] (see Supplementary information) to simulate spatially-varying blur applied to ground truth images from Google’s Open Images dataset [37], [38]. This enables efficient and accurate generation of large training datasets with spatially varying PSFs, a task that would otherwise be prohibitively slow or experimentally impractical. Each simulated blurred image is corrupted with noise by augmenting a measured flatfield through random rotations and flips (Figure 3b). The total time taken to simulate 3,500 noisy and blurred images on a single NVIDIA L40 GPU was approximately 10 min.

A Restormer network [40] is trained using these 3,500 simulated images as input, and their corresponding ground truth images as the desired output (Figure 3c). We use the default parameters and loss functions described in the original paper [40] for the Restormer network, except reducing the number of channels to [36, 72, 144, 288] for layers L1 to L4, respectively due to GPU memory constraints. This constitutes a total of 14.8 million trainable parameters in our Restormer network. Using 4 NVIDIA L40 GPUs, training for 200 epochs with a batch size of 1 took approximately 48 h.

**Figure 4:**
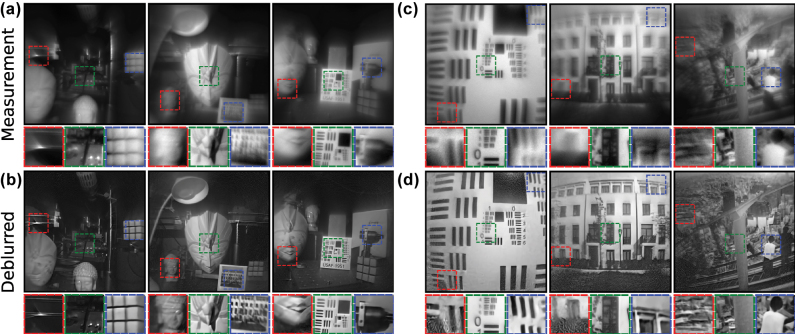
Deblurring images from the hyperbolic metalens camera using the trained, reference-free Restormer network. The (a) measured and (b) deblurred images of scenes taken around a lab. The (c) measured and (d) deblurred images of printed photos placed before the camera. All images have the same angular FOV of 54°.

Figure 4 shows the raw measurements from our hyperbolic metalens camera and the corresponding results of Restormer deblurring on the images (see Supplementary information for more results). The characteristic aberrations due to the hyperbolic lens phase profile are evident in the sharp features at the center of the image and the increasing coma at larger incidence angles. The images here are not diffraction-limited due to the broadband LED used to illuminate the scenes and photos. The physical size of the detector pixels also limits the resolution of the measurements.

**Figure 5:**
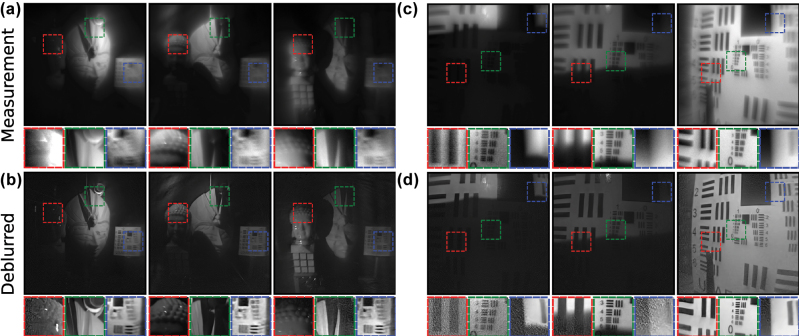
Deblurring images from the hyperbolic metalens camera with varying illumination direction and obstructions. The (a) measured and (b) deblurred images of the same lab scene under different lighting. The (c) measured and (d) deblurred images of a printed USAF card, where the card was tilted in the last column. All images have the same angular FOV of 54°.

Despite the spatially-varying aberrations in the measurements, the trained Restormer network is able to deblur the full FOV of the images in real-time (∼50 ms per image), recovering features even toward the edges of the images. By using a reference-free dataset, we avoid overfitting to specific imaging conditions during the training of the Restormer network. This is further demonstrated in Figure 5 where under varying illumination directions and obstructions, our trained Restormer is still able to recover features even with low lighting at various regions of both the scene and printed USAF card.

**Figure 6:**
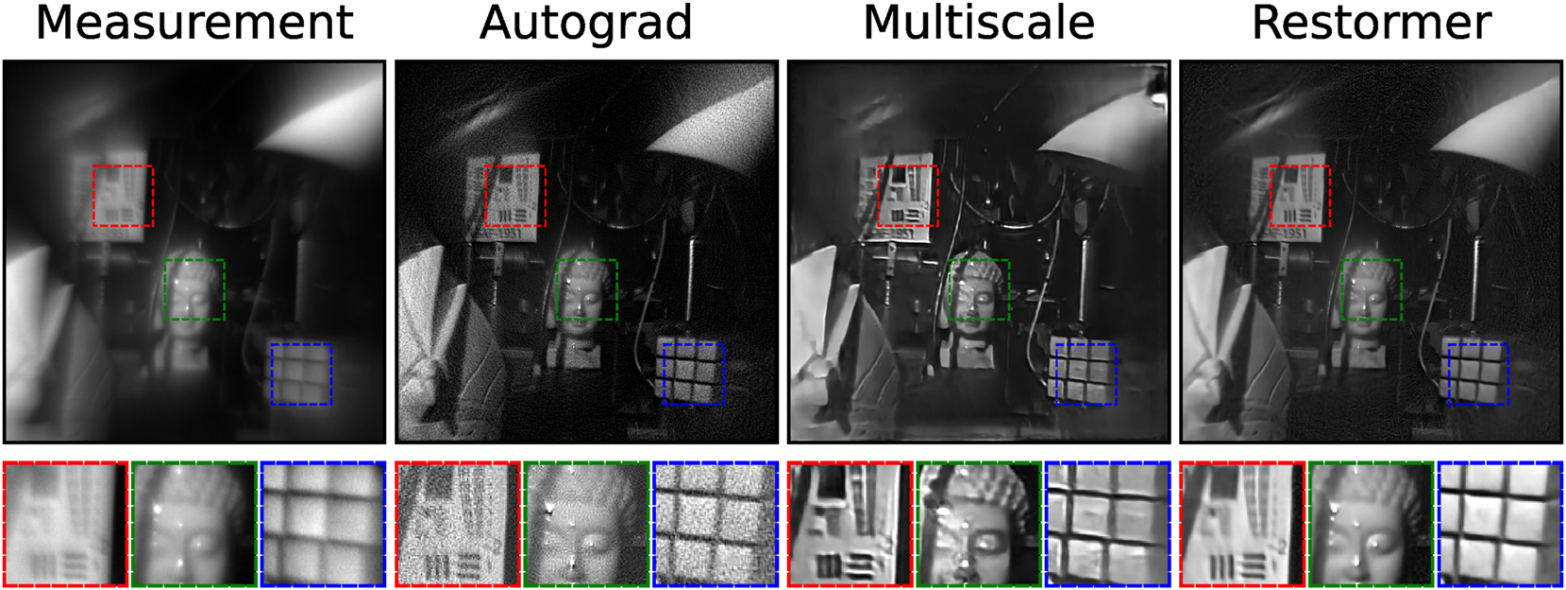
Comparing deblurring performance using various algorithms. The Restormer network surpasses both Autograd and Multiscale methods in both spatially-varying deblurring capabilities as well as suppressing noise.

Figure 6 further demonstrates the improved quality of deblurring from our trained Restormer network over other existing state-of-the-art approaches. The first of which is an Autograd implementation of the eigenCWD algorithm [26] which utilizes PyTorch’s [41] inbuilt automatic differentiation engine to perform optimization instead of using analytical gradients (details in Supplementary information). The reconstruction from this iterative approach in Figure 6 is contaminated with noisy artifacts as it only accounts for spatially-varying blur and not the noise characteristics of the sensor. In addition, as an iterative algorithm, the Autograd implementation of eigenCWD is incapable of real-time deblurring (∼1 min per image on a single NVIDIA L40 GPU). Using the same dataset, we also trained a Multiscale neural network architecture [42] which has recently been used in image reconstruction applications for metalenses [31], [32] (details in Supplementary information). However, we observe residual smeared artifacts in the output of the Multiscale network, likely attributed to the presence of noise in the training dataset which the network is unable to fully remove. This suggests that the Restormer network remains robust against noise and demonstrates improved performance in spatially-varying deconvolution over existing state-of-the-art methods.

## 3 Conclusions

In this work, we have demonstrated wide FOV imaging with a hyperbolic metalens camera. By using the eigenPSF method as an efficient forward model to simulate the metalens’ spatially-varying blur computationally, we circumvent the need for experimental curation of datasets for training a deblurring neural network. In addition, the Restormer network used for postprocessing the images enables real-time aberration correction (after training) compared to time-consuming iterative algorithms, and additionally remains robust against noise and experimental errors.

Our findings suggest that the FOV in hyperbolic metalens imaging could be further extended by leveraging advances in computational power to train on larger image sizes. Additionally, the diffraction-limited resolution of the hyperbolic lens along the optical axis remains underutilized due to current limitations in detector pixel sizes and the large bandwidth of the illumination source. With future improvements in hardware, this work has the potential to open new pathways toward achieving high-resolution, wide-FOV imaging with hyperbolic metalenses.

## 4 Methods

### 4.1 Fabrication of metalens

A 193 nm argon fluoride (ArF) deep-ultraviolet (DUV) immersion photolithography process combined with a dry etching process is used to fabricate the hyperbolic metalenses. The metalens comprises millions of amorphous silicon (a-Si) nanopillars, which are patterned on a 350 nm-thick a-Si film deposited using plasma-enhanced chemical vapor deposition (PECVD) on a 12-inch fused silica wafer. The wafer was then diced into small coupons, and the individual a-Si metalenses were subsequently etched using a dry etching process. Hence, the metalens pattern was transferred from the photoresist to the a-Si film, forming a-Si nanopillars. The 480 nm a-Si pillars were etched in multiple smaller steps instead of a continuous step. This process is recommended, especially when performing deep etching with high aspect ratio pillars. After every etching step, the chamber undergoes a 5-min cooling period before the next step. This not only provides smoother sidewalls but also protects the pillars from undercutting. A residue layer of SiO_2_ (30 nm) remains on top after a-Si etching as a part of the etching hard mask, but it possesses no hindrance to the optical performance, and therefore, we do not remove it.

## Supplementary Material

Supplementary Material Details
