# Supplementary material for: Neural network enabled wide field-of-view imaging with hyperbolic metalenses
Source: Nanophotonics. 2025 Sep 18;14(20):3329–37. doi: 10.1515/nanoph-2025-0354 (PMC12588562; doi:10.1515/nanoph-2025-0354)
Supplement: Supplementary file 1 — Supplementary Material Details [file j_nanoph-2025-0354_suppl_001.pdf]

Supporting Information for neural network enabled wide field-of-view imaging with hyperbolic metalenses

Joel Yeo[1,2,3], Deepak K. Sharma[3], Saurabh Srivastava[3], Aihong Huang[3], Emmanuel Lassalle[3], Egor Khaidarov[3], Keng Heng Lai[5], Yuan Hsing Fu[5], N. Duane Loh[1,2,4], Arseniy I. Kuznetsov[3,*], and Ramon Paniagua-Dominguez[3,+]

[1]NUS Graduate School for Integrative Sciences and Engineering Programme, National University of Singapore, 119077, Singapore
[2]Department of Physics, National University of Singapore, 117551, Singapore
[3]Institute of Materials Research and Engineering (IMRE), Agency for Science, Technology and Research (A*STAR), 2 Fusionopolis Way, Innovis #08-03, Singapore 138634, Republic of Singapore
[4]Department of Biological Sciences, National University of Singapore, 117557, Singapore
[5]Institute of Microelectronics (IME), Agency for Science, Technology and Research (A*STAR), 2 Fusionopolis Way, Innovis #08-02, Singapore 138634, Republic of Singapore
[*]Arseniy_Kuznetsov@imre.a-star.edu.sg
[+]Ramon_Paniagua@imre.a-star.edu.sg

1 Fourier optics simulation of PSFs for characterization

The measured PSFs to characterize the fabricated hyperbolic lens in Fig. 1 have a calculated magnification of 83.3. The detector has a reported pixel size of 3.45 µm, which implies that the measured PSFs have an effective pixel size of $\frac{3.45\,\mu\text{m}}{83.3} = 41.4$ nm. However, because it is computationally expensive to simulate the large lens diameter of $d = 5\,\text{mm}$ with such a small pixel size, we choose to simulate PSFs which have a larger pixel size of 165.6 nm, and compare them to the 4x binned versions of the measured PSFs, and these are the depicted images in Fig. 2.

The hyperbolic phase profile is defined as

$$\phi(\mathbf{x}) = \frac{2\pi}{\lambda_0}\left(f - \sqrt{x^2 + y^2 + f^2}\right) \tag{S1}$$

where $\mathbf{x} = (x, y)$ are the cartesian coordinates, and $\lambda_0 = 850\,\text{nm}$ and $f = 1.731\,\text{mm}$ are the designed wavelength and focal length of the metalens. This phase profile is sampled on a 10001×10001 grid with an initial pixel size of 500 nm. The circular lens function is therefore

$$L(\mathbf{x}) = \begin{cases} \exp[i\phi(\mathbf{x})], & \text{for } \sqrt{x^2 + y^2} \leq \frac{d}{2} \\ 0, & \text{otherwise,} \end{cases} \tag{S2}$$

We simulate a plane wave, ψ_0, as

$$\psi_0(\mathbf{x}) = \exp[ik(x \sin\theta_x + y \sin\theta_y)], \tag{S3}$$

where $k = \frac{2\pi}{\lambda}$ is the wavenumber of the plane wave (can be different from λ_0), and θ_x and θ_y are the angles of incidence with respect to the x and y axes.

This plane wave passes through the metalens, which results in the exitwave, ψ_{exit}:

$$\psi_{\text{exit}}(\mathbf{x}) = \psi_0(\mathbf{x}) L(\mathbf{x}) \tag{S4}$$

This exitwave is then propagated to the detector plane at a distance f away to form the PSF using the scaled band-limited angular spectrum (BLAS) method [1]

$$\text{PSF}(\mathbf{x}) = \left|\mathcal{P}_f^m\{\psi_{\text{exit}}(\mathbf{x})\}\right|^2, \tag{S5}$$

where $\mathcal{P}_f^m$ is the BLAS propagator with a magnification of $m \approx 3.019$ such that the final pixel pitch of the simulated PSF is $\frac{500\,\text{nm}}{3.019} \approx 165.6\,\text{nm}$.

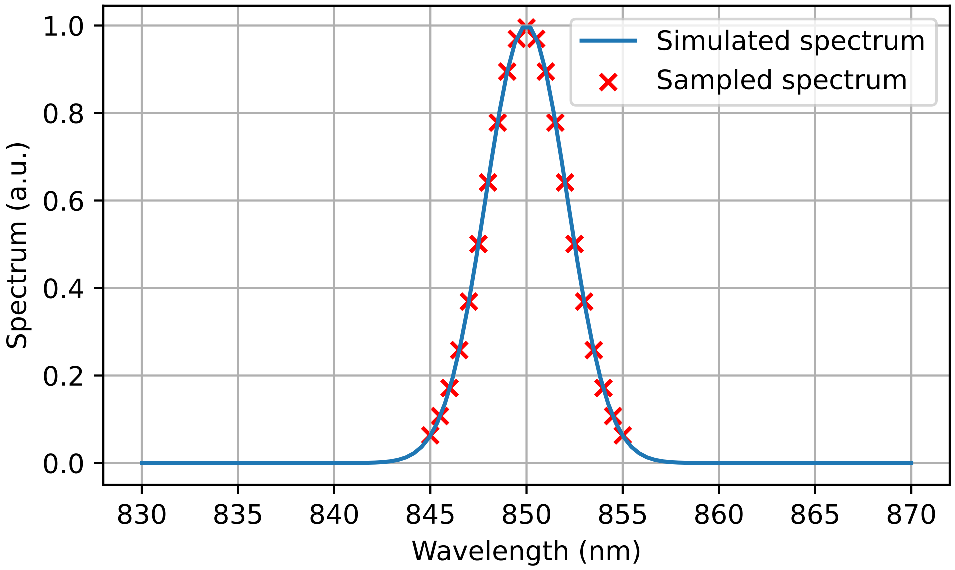

Figure S1: The simulated Gaussian spectrum of the laser with FWHM of 5 nm, and the sampled points used to calculate the broadband PSF.

The laser used to characterize the PSFs has a full width-half-maximum (FWHM) of 5 nm (Fig. S1). We approximate the laser's spectrum as a Gaussian function centered on 850 nm with a standard deviation of $\frac{5\,\text{nm}}{2.355} = 2.123\,\text{nm}$, where the relationship between the FWHM and standard deviation, σ, of a normal Gaussian function is

$$\text{FWHM} \approx 2.355\sigma. \tag{S6}$$

We then sampled 21 equally spaced points from this spectrum between $[845\,\text{nm}, 855\,\text{nm}]$, and the broadband PSF is simply the incoherent sum of these monochromatic PSFs weighted by their corresponding spectrum value.

2 Simulating spatially-varying blur with eigenPSF

Here, we briefly describe the eigenPSF method used to efficiently simulate spatially-varying aberrations. The full details of this method can be found in [2] where open-source code is also available.

For an incoherent imaging system where the point spread function varies as a function of spatial positions, the image formation model is

$$g(x,y) = \iint f(u,v)p(u,v,x-u,y-v)\,\mathrm{d}u\,\mathrm{d}v\,, \tag{S7}$$

where g is the aberrated image, (x, y) denotes the spatial coordinates of the image plane, f is the object, (u, v) denotes the spatial coordinates of the object plane, and p are the corresponding point-spread functions (PSFs) of the imaging system which vary depending on the point emitter's (u, v) coordinates. Explicitly computing the double integral in Eq. (S7) is highly inefficient and slow, and scales like $\mathcal{O}(N^4)$ for an object of size $N \times N$ pixels. In addition, it necessitates experimentally measuring the PSFs at every pixel position, $p(u, v)$, a process that is both time-consuming and susceptible to experimental errors.

Instead, one can approximate the spatially-varying PSFs as a weighted, linear sum of eigenPSFs

$$p(u,v,x,y) = \sum_{i=1}^{\infty} a_i(u,v)q_i(x,y), \tag{S8}$$

where q_i are the eigenPSFs and a_i are the eigencoefficients which encode the variation of the PSFs over the extent of the object. Substituting Eq. (S8) into Eq. (S7) results in

$$g(x,y) = \sum_{i=1}^{\infty} [(fa_i) \otimes q_i](x,y), \tag{S9}$$

where $\otimes$ denotes a convolution operator. Eq. (S9) describes a sum of 2D convolutions, which can be efficiently computed with Fourier transforms based on the convolution theorem with time-complexity of $\mathcal{O}(N^2 \log N)$.

The eigenPSFs, q_i, and eigencoefficients, a_i, are numerically calculated by performing an eigendecomposition on a stack of PSFs at various sampled (u, v) locations on the object plane. The mathematical details can be found in [2].

In this paper, we experimentally measured hyperbolic PSFs at 0°, 1°, 2°, 3°, 4°, 5°, 6°, 7°, 8°, 9°, 10°, 12°, 14°, 16°, 18°, 20°, 25°, 30°, and 40° angles of incidences. These PSFs were numerically rotated to fully cover the finite extent of the imaged object (see Fig. 3), resulting in a total of 692 PSFs. We then computed the corresponding eigenPSFs and eigencoefficients from these 692 PSFs and used Eq. (S9) to simulate the spatially-varying aberrated images for our training dataset.

3 Modulation transfer function of the hyperbolic metalens

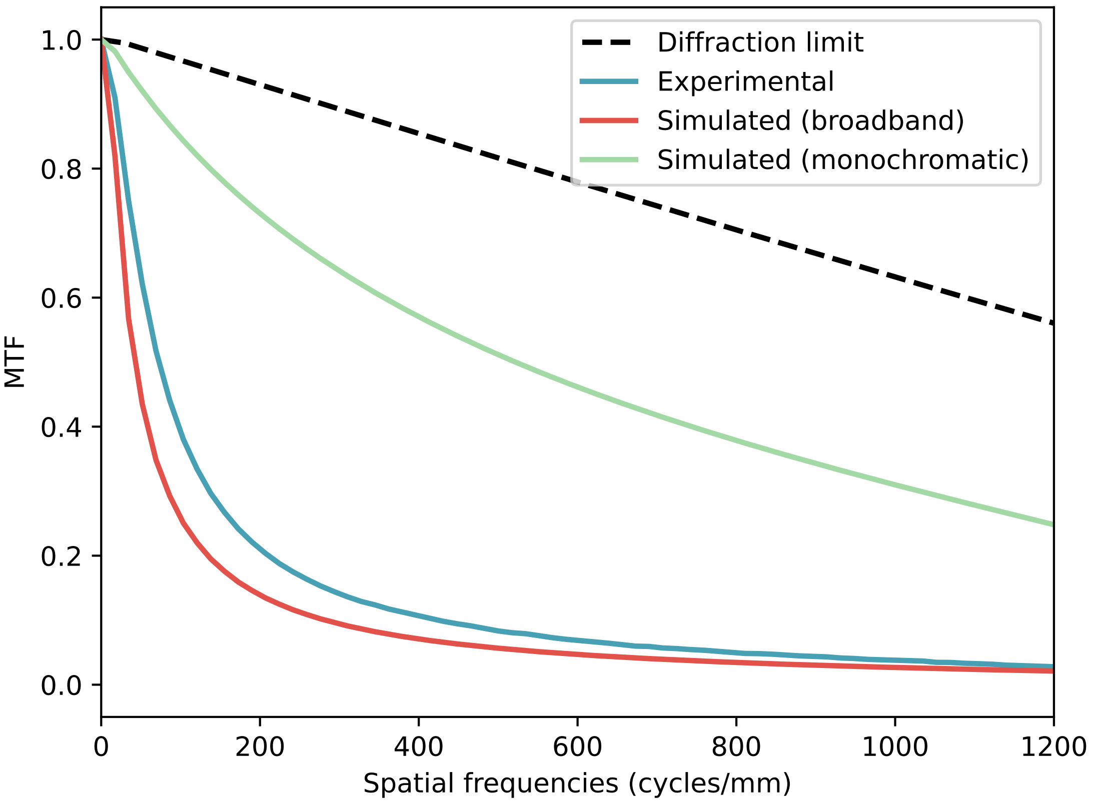

Figure S2: Experimental and simulated modulation transfer functions (MTF) of the hyperbolic metalens at 0° angle of incidence. The significant deviation from the diffraction limit is due to the broadband nature of the laser (FWHM $\approx 5\,\mathrm{nm}$) used to measure the PSFs.

4 Imaging setup for hyperbolic metalens camera

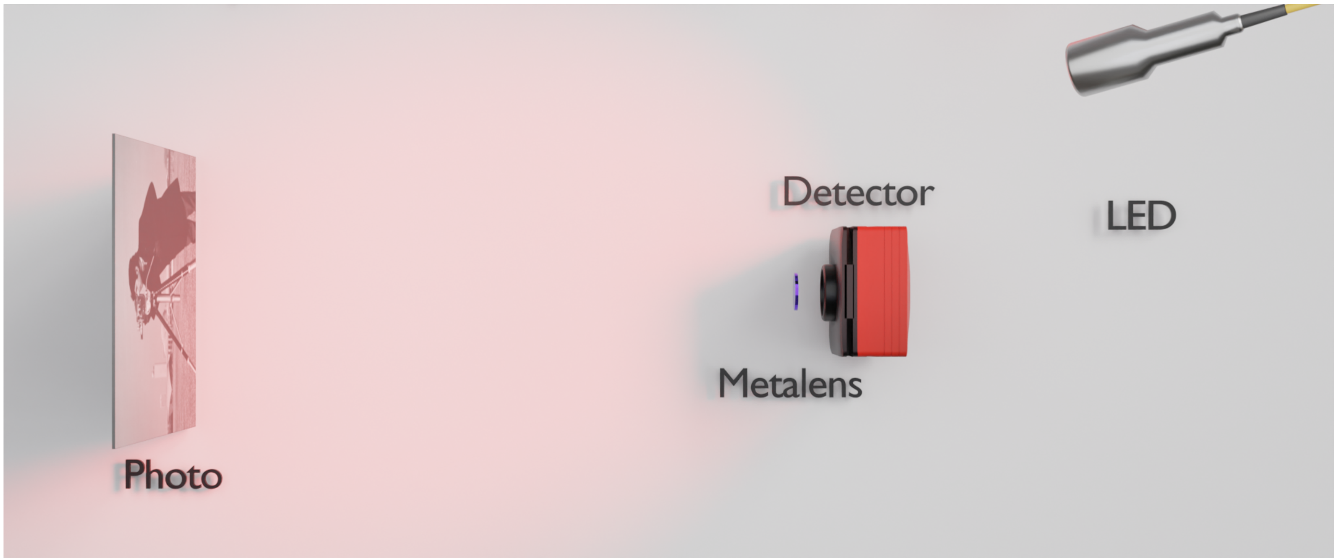

Figure S3: Imaging setup for the hyperbolic metalens camera. The scene is illuminated with an LED with a dominant wavelength of 850 nm (Thorlabs M850L3) and bandwidth of 30 nm. The detector's outer casing is removed (not depicted here) to allow for direct mounting of the metalens a focal length away from the sensor (see Fig. 1g,h of the main manuscript).

5 Comparison of deblurring algorithms on simulated images

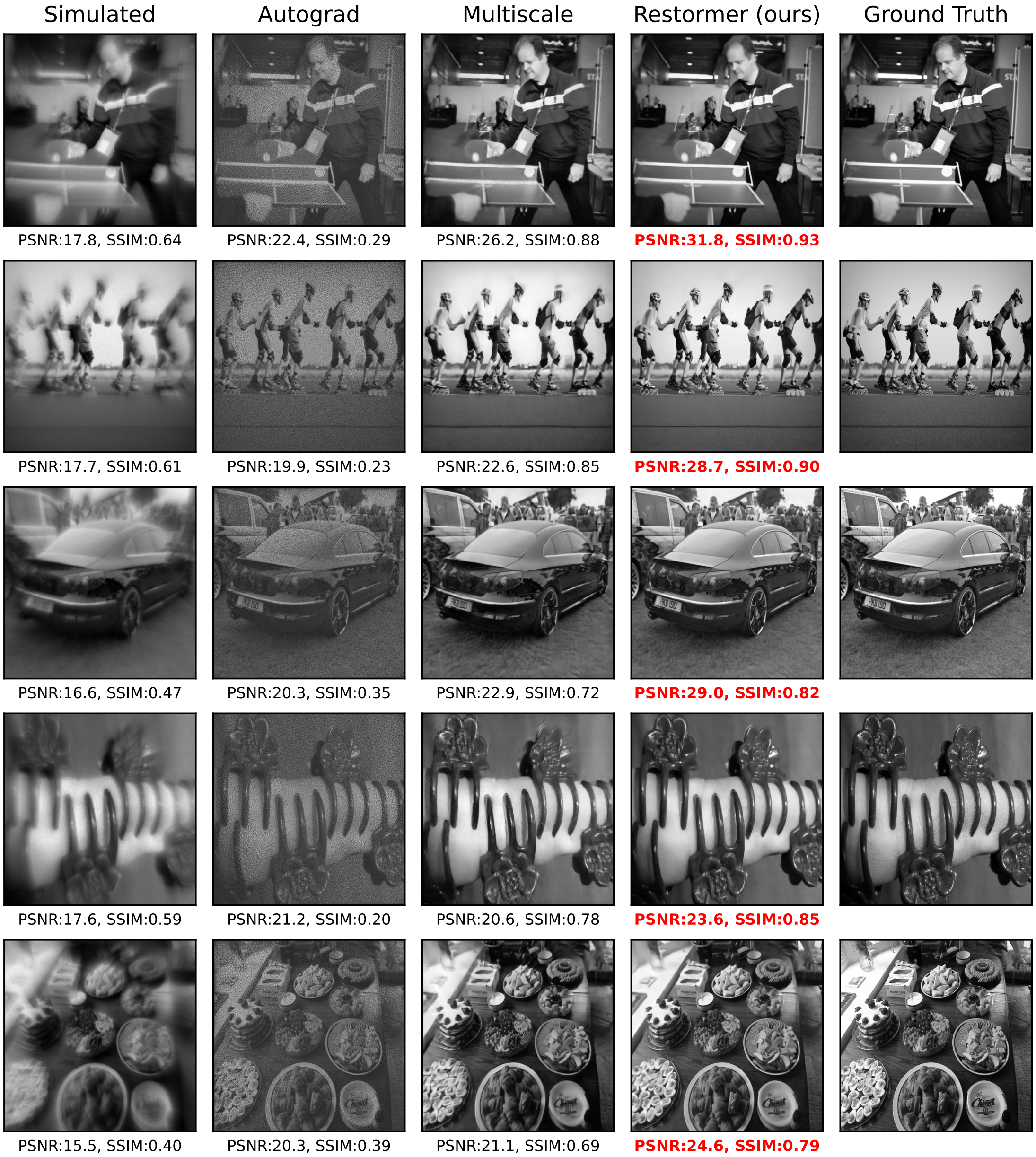

Figure S4: Comparison of deblurring algorithms on simulated images. The deblurred images' peak signal-to-noise ratio (PSNR) and structural similarity index measure (SSIM) are computed against the ground truth. The Restormer consistently achieves the highest PSNR and SSIM.

6 Restormer deblurring of experimental and simulated images

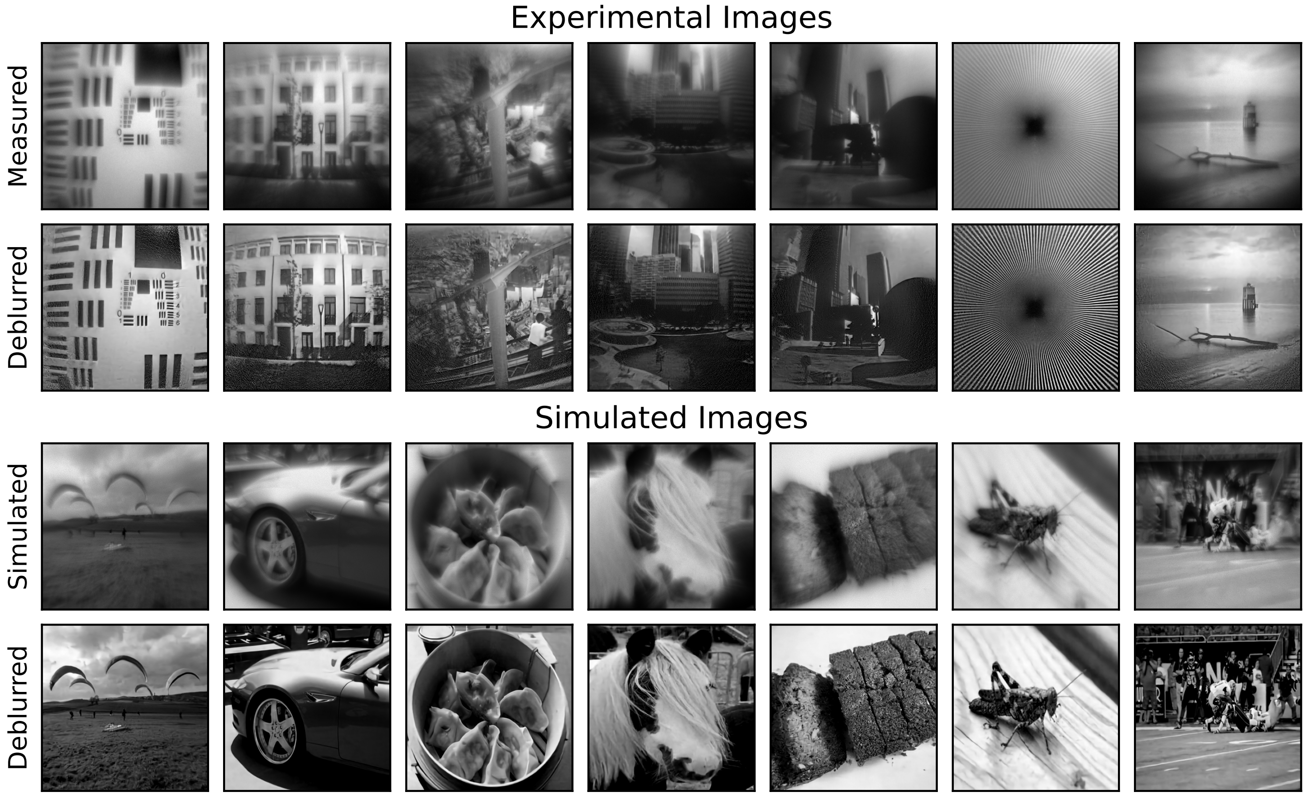

Figure S5: Restormer deblurring for both experimental and simulated images.

7 Autograd implementation of the eigenCWD algorithm

The eigenCWD algorithm [2] seeks a solution that minimizes the following objective function based on the eigenPSF forward model described in Eq. (S9):

$$\min_{\mathbf{f}} E(\mathbf{f}) = \min_{\mathbf{f}} \frac{\mu}{2} \|\mathbf{g}_{\text{eigenPSF}}(\mathbf{f}) - \mathbf{g}_{\text{measured}}\|_2^2 + \alpha \|\nabla \mathbf{f}\|_1, \tag{S10}$$

where E is the loss function, $\mathbf{f}$ and $\mathbf{g}$ denote the 2D-array representation of the object and blurred image respectively, μ and α are hyper-parameters, and $\nabla \mathbf{f}$ is the total variation regularizer applied on the object. In the original implementation, the authors solve Eq. (S11) using the alternating direction method of multipliers (ADMM) method which required analytical gradients to be calculated. This makes it tedious to change the loss function and regularizer as the analytical gradients have to be recalculated again.

Instead, we utilize PyTorch's automatic differentiation engine which uses the chain rule to computationally calculate the gradient update based on the differentiable, elementary functions used in the forward model of Eq. (S9).

This presents a highly flexible optimization method as one can easily change the loss metric or regularization function without the need to recalculate analytical gradients.

In the example shown in Fig. 5 of the paper, we used the following loss function:

$$\min_{\mathbf{f}} E(\mathbf{f}) = \min_{\mathbf{f}} \mathrm{SSIM}(\mathbf{g}_{\mathrm{eigenPSF}}(\mathbf{f}),\, \mathbf{g}_{\mathrm{measured}}), \tag{S11}$$

where the SSIM is the structural similarity index measure [3]. We use PyTorch's inbuilt stochastic gradient descent (SGD) optimizer with default parameters and a learning rate of 100. The code for this Autograd implementation of eigenCWD is available from the authors upon reasonable request.

8 Multiscale Neural Network

In this paper, we used the default parameters of the original Multiscale architecture [4], except for changing the convolution kernel size to 7×7, and the error metric from mean-squared error (MSE) to SSIM. Through our empirical tests, these changes led to improvements in reconstructions. We trained with a batch size of 64 for a total of 1000 epochs, which took approximately 7 hours to complete on 4 NVIDIA L40 GPUs.

References

[1] Xiao Yu, Tang Xiahui, Qin Yingxiong, Peng Hao, and Wang Wei. Band-limited angular spectrum numerical propagation method with selective scaling of observation window size and sample number. *J. Opt. Soc. Am.*, 29(11):2415, November 2012.

[2] Joel Yeo, Duane Loh, Ramón Paniagua-Domínguez, and Arseniy Kuznetsov. EigenCWD: a spatially-varying deconvolution algorithm for single metalens imaging. *Opt. Express*, November 2024.

[3] Zhou Wang, Alan Conrad Bovik, Hamid Rahim Sheikh, and Eero P Simoncelli. Image quality assessment: from error visibility to structural similarity. *IEEE Trans. Image Process.*, 13(4):600–612, April 2004.

[4] S Nah, T Hyun Kim, and K Mu Lee. Deep multi-scale convolutional neural network for dynamic scene deblurring. In *Proceedings of the IEEE conference on computer vision and pattern recognition*, pages 3883–3891, 2017.
